# Development of intrasentential code-switching patterns in Spanish-speaking English learners

**DOI:** 10.1093/chidev/aacaf059

**Published:** 2026-05-06

**Authors:** Julie M Schneider, Giovanna Morini, Veronica De Jesus, Aquiles Iglesias

**Affiliations:** The University of California, Los Angeles, UC|CSU Collaborative for Neuroscience, Diversity, and Learning, School of Education and Information Studies, 457 Portola Plaza, Los Angeles, CA 90095, United States; Communication Sciences and Disorders Department, The University of Delaware, 100 Discovery Blvd., Newark, DE 19713, United States; Communication Sciences and Disorders Department, The University of Delaware, 100 Discovery Blvd., Newark, DE 19713, United States; Communication Sciences and Disorders Department, The University of Delaware, 100 Discovery Blvd., Newark, DE 19713, United States

**Keywords:** English learners, code-switching, language proficiency

## Abstract

Bilingual children often engage in code-switching (CS) or switching between languages. Research indicates CS shifts as a child's L1 and L2 proficiency change, yet little is known about how these patterns unfold in structured academic tasks. This study examined CS in 195 Spanish-speaking English learners on narrative recall tasks in Spanish and English from kindergarten through second grade (4;9–9;1; 57.9% female). In kindergarten, children switched into Spanish on the English task, but by second grade, they switched into English on the Spanish task. The patterns of CS paralleled shifts in relative proficiency: weaker skills in English predicted switching into Spanish, whereas weaker skills in Spanish predicted switching into English. These findings highlight CS as a dynamic reflection of developing bilingual proficiency.

Bilingual children show a wide variation in their first (L1) and ­second (L2) language skills when they enter school (Su et al., 2024), and their proficiency in each language continues to shift over time (Castilla-Earls et al., 2019; Duran et al., 2023; Hoff & Core, 2013; Rojas & Iglesias, 2013). These dynamic trajectories have been attributed to variation in the quantity and quality of the languages used by peers, schools, and communities (Adamson et al., 2021; Hirsh-Pasek et al., 2015; Paradis & Grüter, 2014; Unsworth, 2016). Importantly, variability is also evident in how children use their linguistic repertoires: during the early school years, bilingual children will often attempt to use all of their linguistic resources in the communicative interactions in which they engage (­Beatty-Martínez et al., 2020; Hereida & Altarriba, 2001; Lipski, 2014; Zentella, 1997), and this overtly appears as if the child is switching from one language to another. The focus of this paper is on these external manifestations of code-switching in bilingual children's spoken language—particularly during structured narrative recall tasks, which are often common in academic settings—without implying the internal mechanism that led to these behaviors (see Garcia & Lin (2017) and MacSwan (2017) for a discussion of code-switching and translanguaging).

Code-switching, or switching between languages, is a common feature of the speech style used in bilingual communities and has been well documented in speakers of different ages—including young bilingual children (Genesee & Nicoladis, 2007; Ribot & Hoff, 2014). Code-switching can be broadly defined as an alternation between two languages during language production (Smolak et al., 2020). It is (i) governed by grammatical constraints (Bhat et al., 2016; Gutiérrez-Clellen et al., 2009; MacSwan, 2016; Paradis et al., 2000; Riegelhaupt, 2000), (ii) can occur both within (intrasentential) and across utterances (intersentential), (iii) can vary across contexts, and (iv) is constrained by a complex network of sociolinguistic variables (Gutiérrez-Clellen et al., 2009; Lipski, 1985; McClure & McClure, 1988; Montanari et al., 2019; Poplack, 1980; Reyes, 2004). In addition to various socio-pragmatic functions (e.g., emphasis of a point, change topic of conversation), code-switching might occur when children try to maintain communicative intent and optimize performance by filling lexical or morphosyntactic knowledge gaps in one language with lexical or morphosyntactic structures from another (Bernardini & Schlyter, 2004; Genesee & Nicoladis, 2007; Greene et al., 2013; Halpin & Melzi, 2021; Raichlin et al., 2019).

## Theoretical models of code-switching

Theoretical models of code-switching have evolved over the years and vary based on production and comprehension processes (Tamargo et al., 2016). The present study focuses on the production of code-switches and serves as a way of expanding on existing theories within this domain. Some of the most accepted models suggest that production patterns in speech containing code-switches are driven by (i) cognitive control abilities, (ii) distributional patterns of the languages at play, (iii) language proficiency of the speakers, and (iv) sociolinguistic factors. One general hypothesis is that bilinguals and second-language learners code-switch, in part, to improve fluency during production (Kroll & Bialystok, 2013). Given that both languages are activated (to some degree) during production, code-switching may be in part the result of speakers trying to mitigate planning difficulties that are linked to parallel activation of the languages. Additionally, the patterns of code-switching observed during speech production (i.e., where the language switch happens in a sentence or across sentences) are thought to be guided by language-specific properties that interact with production mechanisms (Costa et al., 1999; Miozzo & Caramazza, 1999; Schiller & Caramazza, 2003; Vigliocco et al., 1996). In other words, a speaker's lexical concepts are organized into a functional structure in which syntactic roles are specified, then these lexical items are assembled into a sentence, and finally, this output is transferred onto a final stage in which phonetic encoding and articulatory processing take place (Bock & Levelt, 1994; Jaeger & Norcliffe, 2009). In the case of code-switching, different words across each language have different properties, which may make them more (or less) likely to be switched from one language to another. One common example is that of auxiliary words in Spanish, where a word like “estar” (to be) is more likely to be code-switched, while a word like “haber” (to possess or to have) is less likely to be code-switched into the other language (Coromines & Pascual, 1980; Myers-Scotton & Jake, 2001). The idea here is that a word like “estar” has a syntactic behavior that is more autonomous, which makes it so that code-switching can occur more effortlessly (e.g., it can be followed by various expressions, intervening words are permissible between “estar” and a present participle, and “estar” and the present participle can switch syntactic positions). A word like “haber”, on the other hand, is much more restricted in how it can be used, which considerably limits the possibility of a code-switch. Therefore, the characteristics of the words within the syntactic context of the languages also contribute to the model. Nevertheless, the fact that the amount of code-switching varies across speakers also suggests that there are factors associated with community-based norms that affect whether or not bilinguals engage in code-switching behaviors, with code-switching being more common (and accepted) in some multilingual communities compared with others (Torres Cacoullos & Travis, 2015).

## Code-switching in preschool and school-age children

The emergence of code-switching occurs from the earliest stages of language production (Meisel, 1994; Paradis et al., 2000; Ribot & Hoff, 2014). By preschool, bilingual children switch systematically between languages based on the setting, interlocutor, message, topic of conversation, and task (Genesee, 2002; Gort, 2012; Gutierrez-Clellen et al., 2009; Halpin & Melzi, 2021; Montanari et al., 2019; Peynircioglu & Durgunoglu, 2002; Vu et al., 2010). Despite differences in methodology, the results of existing code-switching studies tend to be rather consistent: not all bilingual children code-switch, however, bilingual children code-switch more when they speak their nondominant language, and although some studies have found comparable rates of code-switching across conversational and structured tasks (e.g., Faroqi-Shah et al., 2022; Gutiérrez-Clellen et al., 2009), others report that children are more likely to code-switch during conversational interactions, where the discourse is more spontaneous and socially contingent (e.g., Lanza, 1997; Raichlin et al., 2019; Wei, 2013; Zentella, 1997). These task-related differences in code-switching may reflect the varying cognitive and social demands placed on children, as narratives often elicit more structured and formal language use, while conversations are more flexible and influenced by the interlocutor, topic, and immediate communicative goals. Thus, considering the elicitation context is critical when interpreting code-switching behavior in bilingual children.

Methodologically, code-switching studies of preschool and school-age children have varied with respect to participants’ age, interlocutors, characteristics of the instructional program in which they were enrolled, and language elicitation methods. ­Gutierrez-Clellen et al. (2009) selected students (mean age 6;1 years) who were attending a Head Start program where instruction was in English and Spanish. Story narratives and conversational samples were used to elicit language samples, and only intrasentential code-switching was examined. The proportion of utterances containing intrasentential code-switches was low in the English narrative and conversational tasks (0.012 and 0.012, respectively) and higher in the Spanish tasks (0.114 and 0.159, respectively). Gross and Kaushanskaya (2022) expanded on this work with children from a wider age range (4;0–6;11 years) using a different elicitation method. Children were enrolled in a variety of programs where instruction was either in Spanish, in English, or both, and completed a scripted dialogue task in which the examiner spoke only Spanish, only English, or both. On this task, children were more likely to engage in intrasentential code-switching when interacting with a Spanish-speaking partner than when interacting with an English-speaking partner. Taken together, these studies highlight how both the language elicitation methods and interlocutors influence patterns of intrasentential code-switching among bilingual children at a single point in time.

Several longitudinal studies have examined how code-switching changes during the preschool years. Kuzyk et al. (2020) found that French–English children code-switched very little between 36 and 61 months, with no developmental change across this period. Smolak et al. (2020) extended this work in Spanish–English dyads in San Diego and French–English dyads in Montreal (31–39 months), showing that code-switching trajectories differed across sociocultural contexts: in San Diego, Spanish-to-English switching remained stable while English-to-Spanish switching declined, whereas in Montreal, English-to-French switching declined. These patterns were attributed to community attitudes and relative language prestige (Smolak et al., 2020). Montanari et al. (2019) examined slightly older dual language learners (ages 3;6–4;5) in a Head Start program and reported that overall code-switching was infrequent but shifted over the year: switching decreased on English tasks, slightly increased on Spanish tasks, and became more intrasentential in Spanish, consistent with research linking intrasentential switching to greater bilingual proficiency (Poplack, 1980; Yow et al., 2016).

Code-switching studies of children older than 3 years of age have focused primarily on preschoolers, a time at which many bilingual children are first systematically exposed to English. Yet one-third of low-income Latino children do not participate in early childhood education, and among those who do, 40% are not in center-based programs (Crosby et al., 2016). For these children, initial exposure to English occurs only upon school entry, and opportunities to use both languages during the school day depend on the program of instruction—an issue which is often not controlled for in studies of code-switching. In English immersion classrooms, Spanish use is limited (Durán et al., 2023; Franco et al., 2019; Sawyer et al., 2018), which may constrain L1 development and accelerate shifts toward English (Castilla-Earls et al., 2019; Collins, 2014; Rojas et al., 2019). Existing longitudinal studies also tend to follow children for short periods, potentially obscuring longer-term developmental trajectories. For example, bilingual children's proficiency in English and Spanish will undergo change and show different trajectories (e.g., greater grammaticality in English and lesser grammaticality in Spanish; Castilla-Earls et al., 2023), and language growth discontinuities are observed across summer breaks (Rojas & Iglesias, 2013). The current study extends prior research by tracking bilingual children from Kindergarten through second grade in English immersion classrooms. By focusing specifically on English learners and examining their narrative code-switching, we aim to capture how developing proficiency in English shapes code-switching across the early school years.

## Relative language proficiency and code-switching

Bilingual children, regardless of whether they are simultaneous or sequential bilinguals, must uncover the unique features of the languages to which they are exposed. Greater exposure to one language provides more opportunities to acquire its vocabulary and morphosyntax, while systematic exposure to a second language in school gradually builds L2 knowledge (Collins, 2014; Hoff & Ribot, 2017). The quantity and quality of input, along with children's internal capacities, determine proficiency in each language (Bedore et al., 2012). Most bilinguals never attain equivalent proficiency in both languages, especially across all communicative tasks (Treffers-Daller, 2011). Instead, proficiency shifts over time, with children typically demonstrating relative strength in one language, and this balance changes as English use increases (Gathercole, 2014). Importantly, these shifts occur unevenly across domains and time points (Peña et al., 2021). Peña et al. (2023) described this as mixed dominance, where semantic and morphosyntactic skills are distributed unevenly across ­languages. This uneven distribution can also emerge within domains (Su et al., 2022, 2024). For example, a child may know more prepositions in English but more conjunctions in Spanish. Such asymmetries create lexical or syntactic gaps that often surface in code-switching productions. As proficiency in one language increases, code-switching to compensate for gaps should decline. Conversely, children are more likely to code-switch when lacking a translation equivalent or grammatical structure in their less proficient language.

In general, results indicate an inverse relationship between language proficiency in a given language and code-switching when speaking that language (Greene et al., 2013; ­Gutiérrez-Clellen et al., 2009; Poulisse & Bongaerts, 1994). For example, Greene et al. (2013) found that English-dominant 5-year-olds, as indicated by reported language exposure and use, code-switched less frequently to Spanish when lexical items were presented in English, as compared with code-switching more frequently to English when lexical items were presented in their weaker language—Spanish. Similarly, Spanish-dominant children code-switch less frequently to English when lexical items were presented in Spanish as compared with more code-switching to Spanish when lexical items were presented in children's weaker language—English. Gutiérrez-Clellen et al. (2009) also found that English-dominant children code-switch less frequently to Spanish when asked to retell a story in English, as compared with a higher number of code-switches to English when the story was elicited in Spanish. On the other hand, in the same study, Spanish-dominant children tested in their weaker language (English) showed a very low frequency of code-switching to their stronger language (Spanish), and they code-switched more frequently from their stronger to their weaker language. Interestingly, children with balanced proficiency across languages can show either high (Yow et al., 2018) or low rates of code-switching (Ribot & Hoff, 2014).

Given that language proficiency plays a critical role in children's code-switching, numerous studies have examined the relationship between the two. However, the way by which proficiency is operationalized varies substantially across studies. Parent and/or teacher reports are oftentimes used to determine (i) the child's use of each language during the day to make inferences about how strong each language is (i.e., language proficiency; Gutiérrez-Clellen et al., 2009) and (ii) the relative strength of each language with respect to the other (i.e., language dominance; Greene et al., 2013; Gutiérrez-Clellen et al., 2009; Ribot & Hoff, 2014). However, these are indirect measures that can be affected by the frequency with which parents and teachers interact with children. Standardized language assessments may be utilized in place of parent and/or teacher reports to measure children's language proficiency within each language. Using semantic subtests of the Bilingual English–Spanish Oral Screener (BESOS, Peña et al., 2018), Greene et al. (2013) measured children's receptive and expressive language skills in each language. They found that children who were more proficient in Spanish were significantly more likely to code-switch on an English task, while children who were more proficient in English were significantly more likely to code-switch on the Spanish task. It is possible though that differences in proficiency are not global but rather specific to expressive, but not receptive, skills. Specifically, bilingual children's expressive skills in their L2 may be delayed in comparison with their receptive skills (referred to as the expressive-receptive gap—Gibson et al., 2012, 2022)—having downstream effects on their code-switching patterns.

In addition to standardized measures of language proficiency, language sample analysis is currently one of the most reliable direct methods for determining language proficiency in bilinguals (MacSwan & Rolstad, 2006), as it allows for the measurement of microlinguistic constructs for diagnostic purposes, such as lexical diversity, grammaticality, and syntactic complexity during discourse (Heilmann et al., 2008; Kapantzoglou et al., 2017). Despite its utility, few studies have relied upon the analysis of language samples to measure language proficiency in more naturalistic contexts. Studies utilizing language sample analysis have focused on two primary aspects of lexical and grammatical proficiency: number of different words (NDW) and mean length of utterances (MLU). MLU is typically used when measuring grammatical productivity across languages with different morphological patterns (Goldstein et al., 2010), while NDW is commonly used as an indicator of children's lexical development across languages (Hewitt et al., 2005). NDW has often been used as a measure of children's lexical development (Hewitt et al., 2005; Thordardottir, 2005; Uccelli & Paéz, 2007; Watkins et al., 1995) and is typically divided by the number of total words (NTW) or duration of the language sample to garner the proportion of different words used relative to children's overall language productivity. It has been reported that children who possess a higher NDW in their L1, but not their L2, are more likely to code-switch (Yow et al., 2011, 2018). However, after entering school, bilingual children experience accelerated growth in their L2 proficiency and a deceleration in their L1 proficiency (Hoff et al., 2018; Ruiz-Felter et al., 2016; Scheffner Hammer et al., 2008), which may differentially influence their likelihood of code-switching.

The second measure, MLU, in words, is the most widely used measure of grammatical productivity (Bedore et al., 2010) and is typically used when comparing languages with different morphological patterns (Goldstein et al., 2010). Specifically, MLU differentials between a bilingual's L1 and L2 can be used to compare bilingual children to one another, and chart changes in proficiency over time (Yip & Matthews, 2006, 2007). Findings related to MLU are mixed; while one study demonstrated no evidence of a relationship between MLU and code-switching in preschool (Montanari et al., 2019), other studies indicate language proficiency as measured by MLU does, in fact, relate to code-switching (Lam & Matthews, 2020; Schmeißer et al., 2016; Yow et al., 2011, 2018). Yow et al. (2011 , 2018) found that children with a longer MLU in their L1 (Mandarin) were more likely to code-switch, regardless of language. An association between MLU and code-switching has also been observed in Cantonese-English bilinguals (Lam & Matthews, 2020) and Catalan-Spanish bilinguals (Arnaus Gil & Jiménez-Gaspar, 2022 ). Across these studies, when children have a longer MLU in one language (often referred to as language dominance), they are more likely to code-switch in the other, nondominant language.

Despite a wealth of research on the factors underlying children's code-switching, most of this research relies on small samples with differing bilingual experiences, with a limited number of studies examining code-switching longitudinally (Kuzyk et al., 2020; Montanari et al., 2019; Smolak et al., 2020 ). Of these longitudinal studies, all focus on preschool-aged children, and therefore do not explore how children who are bilingual use code-switching to support and extend their conversational and literacy goals onto more structured tasks in school. To our knowledge, the present study is the first that attempts to address these issues by examining the contributions of both NDW and MLU on bilingual children's code-switching patterns during the re-telling of narratives in each of their languages (i.e., Spanish and English), in a relatively large sample of children and over the course of their first 3 years in school. We tested performance on this retell task at six time points: once in the Fall and once in the Spring in Kindergarten, First Grade, and Second Grade. Our original intent was to examine intersentential and intrasentential code-switching; however, preliminary analysis of our transcripts indicated that intersentential code-switching was extremely rare, occurring in only four transcripts of kindergarten children while doing the English task. Therefore, our study focused solely on intrasentential code-switching. From this rich dataset, the current study seeks to answer the following questions: (i) How does code-switching vary across languages as children progress through waves (i.e., Fall vs. Spring terms in school) and grades (i.e., Kindergarten, First, Second)? and (ii) Does language proficiency account for variability in code-switching across languages? Addressing these questions will provide a more comprehensive understanding of what drives code-switching patterns on a more structured task over time in the same population of bilingual children.

## Method

### Participants

Participants were selected from a larger project that investigated the potential sources of variability in literacy skills among children living in the US whose first language was Spanish and were acquiring English as their second language. The project included 1,723 participants from 35 schools in California (Los Angeles and Orange County areas) and Texas (Austin, Houston, and Brownsville). Children had to meet the following inclusionary criteria: (i) designated by the school district as an English learner (EL); (ii) attended a school in which at least 40% of the school population was Latino; (iii) attended a school in which at least 30% of the kindergarten population was identified as Limited English Proficient, as designated by district-based, language proficiency testing; (iv) had never received nor were receiving special education services; and (v) were enrolled in specific programs/models of bilingual instruction (e.g., structured English immersion; transitional bilingual). The proportion of participants across program type was consistent across waves of observation. At school entry (Fall of kindergarten), 42.3% of participants’ mothers voluntarily completed a survey to gauge relative language use (1 = only Spanish, 2 = mostly Spanish, 3 = Spanish and English equally, 4 = mostly English, 5 = only English) at home. The mean relative language use at home in this larger sample was 1.49 (*SD* = 0.81), indicating that family members and peers communicated with their children mostly in Spanish. Narrative language samples were collected twice a year (October and May) over 3 years (within the time window of Fall 2000 through Spring 2005), totaling six waves of data collection between the Fall of kindergarten and the Spring of second grade. Data were collected in both the Fall and Spring of kindergarten, first grade, and second grade. Language sampling occurred during October and May to ensure that participants had been exposed to at least 1 month of academic instruction. For a complete description of the parent database, see Rojas and Iglesias (2013).

The data for the present study were based on a subset of participants who were enrolled in English Immersion programs and had produced narrative samples in English and Spanish at each of the six waves. The final dataset consisted of 2,340 narrative retell language samples (1,170 in each language) produced by 195 typically developing Spanish-speaking ELs in kindergarten through second grade (4;9–7;1 years—kindergarten; 5;9–8;1 years—first grade; and 6;9–9;1 years—second grade). In this sample, 113 children were female, and 82 were male. The mean relative language use at home in this subset was 2.20 (*SD* = 0.84), indicating that children in this subset experienced a higher English language use than the larger sample. The criteria used to select participants for this study (e.g., a minimum of 10 intelligible utterances in each language) might have excluded children with lower English skills. The work of Su et al. (2022) revealed significant heterogeneity in English learners, with one of the profiles for kindergarten children showing stronger English skills. Although one could consider these children to be “English dominant”, their English skills were not sufficient to adequately participate in a regular English class.

### Procedure

#### Narrative language sampling protocol

Narrative language samples in Spanish and English were elicited from the participants using a story retell task of Mercer Mayer's wordless books (Mayer, 1969, 1974, 1975a, 1975b). The work of Heilmann et al. (2016) suggests that negligible differences in language measures were observed across narratives elicited by these four books, with Frog Goes to Dinner providing a slightly higher NDW than the other stories. To promote task familiarity in the native language of the children, sessions were first administered in Spanish. The data collection procedure was replicated in English, using the same story, approximately a week after the first session. Data collectors were fully bilingual (English–Spanish), and different data collectors elicited the narrative samples in each language. The samples were recorded onto digital media and transcribed orthographically using the Systematic Analysis of Language Transcripts Research 2010 (SALT; Miller & Iglesias, 2010), following conventions for bilingual language samples, including segmenting utterances as modified communication units. All instances of code-switching, except for code-switches within mazes and parenthetical comments, were coded at the word and utterance level.

All analyses were based on complete and intelligible utterances, which, as defined by SALT 2010, excluded utterances with unintelligible segments, utterances that were abandoned or interrupted, and nonverbal utterances. The mean number of complete and intelligible utterances in English was 36.96 (*SD* = 12.74) and 35.61 (*SD* = 11.82) in Spanish. Transcription reliability on the original database was conducted at random on 20 English and 20 Spanish samples for protocol accuracy (and transcription accuracy). Protocol accuracy measures ranged from 98 to 100% in English and 94 to 99% in Spanish, and transcription accuracy values on orthographic transcriptions ranged from 90 to 98% in English and 91 to 99% in Spanish. Reliability of code-switching coding was conducted on 40 randomly selected transcripts in English and 40 in Spanish. Reliability ranged from 99 to 100%. This reliability values are standard in the field, as similar coding schemes applied by Heilmann et al. (2008), demonstrated consensus coding of English and Spanish language samples to result in transcription accuracy across coders ranging from 95 to 99%, and to have no significant differences on any of the standard English or Spanish measures used in the current study (i.e., NDW and mean length of utterance).

#### Measures of code-switching and child expressive language skills

Measures of code-switching behavior and expressive language skills were extracted from the transcripts using SALT 2020 (Miller & Iglesias, 2010). A code-switch is defined here as the insertion of a word, phrase, or clause from the nontarget language within an otherwise monolingual utterance (Poplack, 1980). For Spanish narratives, English intrusions were coded as code-switches; for English narratives, Spanish intrusions were coded as code-switches. Repeated interjections, proper nouns, and cognates (e.g., animal, hospital) were not considered code-switches, consistent with prior work (e.g., Rojas & Iglesias, 2013). The outcome measure was the proportion of utterances containing a code-switch, calculated by dividing the number of utterances containing a code-switch by the total number of intelligible utterances. This proportion allows for standardized comparisons across children and contexts.

In addition to the total number of code-switches at the word and utterance level, the NDW children produced, and their MLU in words were selected to quantify expressive language skills of the participants, within each language. NDW was calculated for each language using only words in that language, excluding code-switches. Consistent with the work of Rojas and Iglesias (2013), MLU (a measure of syntactic complexity) was calculated using all of the words in the utterance, regardless of language, as doing otherwise would underestimate the syntactic skills of participants who code-switched due to lexical constraints. Raw values for NDW and MLU within each language, as well as average proportion of code-switches, are provided in Table 1.^1^

**Table 1:** Raw values for proportion of code-switching and child expressive language skills within each language across grades.

		Kindergarten		First grade		Second grade	
		English	Spanish	English	Spanish	English	Spanish
*N*		195		195		195	
Age in months	Mean [range]	69.64 [59–89]		82.03 [71–104]		93.65 [83–112]	
Gender	*N* female (%)	113 (57.9)		113 (57.9)		113 (57.9)	
Total proportion of utterances with a code-switch	Mean (*SD*)	0.05 (0.11)	0.02 (0.05)	0.01 (0.05)	0.02 (0.05)	0.01 (0.02)	0.02 (0.04)
Number of different words (within each language)	Mean (*SD*)	62.97 (23.18)	65.00 (21.14)	83.88 (22.00)	82.27 (21.01)	98.32 (21.23)	87.91 (17.97)
Mean length of utterance (within each language)	Mean (*SD*)	6.20 (1.28)	5.30 (1.13)	6.83 (0.85)	5.86 (0.89)	7.48 (1.20)	6.47 (1.18)

## Results

Given the complex nature of the dataset and the novelty of our research questions, a flexible analytic approach was employed. The models tested were informed by theoretical considerations but not pre-specified and should therefore be interpreted as exploratory, intended to uncover potential relationships rather than test confirmatory hypotheses.

### How does code-switching vary across languages as children progress through waves (i.e., Fall vs. Spring terms in school) and grades (i.e., Kindergarten, First, Second)?

Our first set of analyses sought to address whether the rates of code-switching that children engage in vary across targeted languages, and whether this behavior changes as children progress from kindergarten through second grade. To evaluate this question, we first examined patterns of code-switching across waves and grades. Similar to previous findings (Gutierrez-Clellen et al., 2009), not all children code-switched; 6 children did not code-switch on either the English or Spanish task at any point in time, 16 children did not code-switch at all on the Spanish task, and 63 children did not code-switch at all on the English task. Overall, there was a decrease in the number of children who code-switched on the English task and an increase in the number of children who code-switched in the Spanish task (Table 2).

**Table 2:** Number of children who code-switched on each task across waves.

	Kinder Fall	Kinder Spring	First Fall	First Spring	Second Fall	Second Spring
English task	66	62	68	22	43	11
Spanish task	68	89	94	46	74	112

We modeled the number of code-switches using a Poisson generalized linear mixed-effects model (GLMM) implemented with the glmer function from the lme4 package (version 1.1-20; Bates et al., 2015) in R (R Core Team, 2025). Code-switching frequency is a count-based outcome that often violates assumptions of normality and homoscedasticity, making GLMMs preferable to linear models in this context (Meteyard & Davies, 2020). The dependent variable, number of code-switches, represented the total number of code-switches, including multiple switches within a single utterance (i.e., intrasentential code-switching). As this count could exceed the number of utterances, the outcome was not appropriately modeled as a binomial proportion. To account for individual variation in speech quantity, we included a log-transformed offset term for total utterances, modeling the rate of code-switching per utterance (Jaeger, 2008).

Fixed effects included gender, language of the task (English vs. Spanish), wave (Fall vs. Spring), and grade (Kindergarten, first, second), along with all two- and three-way interactions. We initially considered a more complex random effects structure, including random slopes for language by subject, but these models resulted in convergence warnings and singular fits, suggesting overparameterization. As such, we retained a random intercept for subject to account for repeated measures, which provided a more stable and interpretable model (Barr et al., 2013; Meteyard & Davies, 2020). Coefficients for both fixed and random effects are reported in Table 3.

**Table 3:** Poisson generalized linear mixed-effects model predicting ­code-switching count.

Fixed effects				
Predictor	Estimate (*b*)	*SE*	*z*	*p*
(Intercept)	−2.98	0.11	−28.03	<.001***
Gender (Male)	0.14	0.15	0.92	.357
Language (Spanish task)	−0.97	0.09	−10.39	<.001***
Wave (Spring)	−0.46	0.07	−6.33	<.001***
Grade 1	−1.33	0.09	−15.03	<.001***
Grade 2	−2.13	0.12	−17.93	<.001***
Spanish × Spring	0.15	0.14	1.10	.272
Spanish × Grade 1	1.25	0.14	9.18	<.001***
Spanish × Grade 2	1.64	0.16	9.96	<.001***
Spring × Grade 1	−0.67	0.16	−4.09	<.001***
Spring × Grade 2	−1.07	0.27	−3.92	<.001***
Spanish × Spring × Grade 1	0.61	0.23	2.69	.007**
Spanish × Spring × Grade 2	1.87	0.31	5.97	<.001***

Random effects		
Group	Variance	*SD*
Subject (Intercept)	0.89	0.94

Model fit statistics	
Statistic	Value
Number of observations	2,339
Number of subjects	195
AIC	7,068.5
BIC	7,149.1
Log Likelihood	−3,520.3
Deviance	7,040.5

*Note*. The model was fit using the Poisson distribution with a log link and included an offset for total utterances (log_Utts). Gender was coded as Female = 0, Male = 1; Language as English = 0, Spanish = 1; and wave as Fall = 0, Spring = 1.

**p < .01, ***p < .001.

To ensure the appropriateness of the Poisson distribution, we compared the final model to a negative binomial model using the glmmTMB package (Brooks et al., 2017). The Poisson model showed better overall fit (lower AIC, better residual diagnostics), indicating no strong evidence of overdispersion and supporting its selection over the more flexible negative binomial alternative. Fixed effect significance was determined using *z*-tests, and 95% confidence intervals for model predictions are reported in all figures and *post-hoc* contrasts.

A significant main effect of language indicated that children code-switched significantly less when completing the task in Spanish (i.e., Spanish to English) than in English (i.e., English to Spanish; *b* = −0.97, *SE* = 0.09, *z* = −10.39, *p* < .001). There was also a significant main effect of wave, with children code-switching less in the Spring compared with the Fall (*b* = −0.46, *SE* = 0.07, *z* = −6.33, *p* < .001). The main effect of grade further indicated that, as children progressed through school, their rate of code-switching significantly declined (first grade: *b* = −1.33, *p* < .001; second grade: *b* = −2.13, *p* < .001).

Several significant interaction effects qualified these patterns. The interaction between language and grade suggests that rates of code-switching in each language change across grades. Specifically, compared with kindergarten, children in first (*b* = 1.25, *p* < .001) and second grade (*b* = 1.64, *p* < .001) code-switched significantly more from Spanish to English during the Spanish task, suggesting a developmental shift in code-switching directionality over time. The interaction between wave and grade further indicates that the general decline in code-switching in the Spring is significantly more pronounced in first (*b* = −0.67, *p* < .001) and second grade (*b* = −1.07, *p* < .001) compared with kindergarten.

Most notably, two significant three-way interactions revealed that the Spring effect was moderated by the language of the task. In first grade, the Spring decline in code-switching was less pronounced during the Spanish task (*b* = 0.61, *p* = .007), while in second grade, this pattern reversed, with children showing significantly more code-switches in the Spring from Spanish to English during the Spanish task (*b* = 1.87, *p* < .001). This indicates a developmental shift such that by second grade, children became increasingly likely to code-switch from Spanish to English during the Spanish task, particularly in the Spring, possibly reflecting growing dominance and experience with English (Figure 1).

### Does language proficiency account for variability in code-switching behavior across languages?

We next sought to clarify whether language proficiency accounts for individual variability in code-switching within each language. To examine whether within-language proficiency predicted code-switching during the English task, we used a Poisson GLMM using the glmmTMB package in R (Brooks et al., 2017). This model accounted for the count-based, non-normally distributed nature of code-switching, which was modeled as the raw number of code-switches per session. We included the log-transformed number of English utterances as an offset term to model the rate of code-switching per utterance (Jaeger, 2008), controlling for individual differences in the amount of speech produced. The fixed effects included gender, grade (Kindergarten, first, second), wave (Fall vs. Spring), English MLU (mean length of utterance), and English NDW. A random intercept for subject was included to account for repeated measures across waves. We considered including random slopes, but a maximal random effects structure (Barr et al., 2013), including slopes for grade, wave, or proficiency measures, resulted in singular fits or convergence issues. Therefore, we retained the simpler random intercept model, which offered better convergence and interpretability without compromising model fit (model diagnostics showed no evidence of overdispersion or poor fit). Residual diagnostics were examined using the DHARMa package, confirming that model assumptions were met. Fixed effect significance was assessed using Wald *z*-tests, and 95% confidence intervals were computed via profile likelihood. All continuous predictors were checked for multicollinearity and scaled where appropriate.

The model revealed several significant predictors of code-switching during the English task (see Table 4 for full results). Compared with kindergarten, children in first grade (*b* = −1.28, *SE* = 0.10, *z* = −12.91, *p* < .001) and second grade (*b* = −1.92, *SE* = 0.15, *z* = −12.87, *p* < .001) were significantly less likely to code-switch from English to Spanish on the English task. Code-switching from English to Spanish also decreased in the Spring compared with Fall (*b* = −0.49, *SE* = 0.09, *z* = −5.57, *p* < .001). A higher NDW in English was associated with fewer code-switches from English to Spanish (*b* = −0.02, *SE* = 0.003, *z* = −7.07, *p* < .001), whereas a higher NDW in Spanish was associated with more code-switches (*b* = 0.009, *SE* = 0.002, *z* = 3.65, *p* < .001). Neither English MLU (*p* = .29) nor Spanish MLU (*p* = .20) significantly predicted code-switching, and there was no effect of gender (*p* = .75). These findings suggest that children's vocabulary diversity in both languages plays a key role in code-switching behavior, even within a single-language task context.

**Table 4:** Poisson generalized linear mixed-effects model predicting code-switching count on the English task based on language proficiency.

Fixed effects				
Predictor	Estimate (*b*)	*SE*	*z*	*p*
(Intercept)	−2.84	0.37	−7.71	<.001***
Gender (Male)	−0.08	0.26	−0.32	.748
Grade 1	−1.28	0.1	−12.91	<.001***
Grade 2	−1.92	0.15	−12.87	<.001***
Spring (vs. Fall)	−0.49	0.09	−5.57	<.001***
MLU (English)	0.06	0.05	1.06	.287
MLU (Spanish)	−0.08	0.06	−1.28	.199
NDW (English)	−0.02	0	−7.07	<.001***
NDW (Spanish)	0.01	0	3.65	<.001***

Random effects		
Group	Variance	*SD*
Subject (Intercept)	2.44	1.56

Model fit statistics	
Statistic	Value
AIC	2,774.5
BIC	2,825.2
Log Likelihood	−1,377.3
Deviance	1,159

*Note*. The model was fit using the Poisson distribution with a log link and included an offset for total utterances (log_Utts). Gender was coded as Female = 0, Male = 1 and wave as Fall = 0, Spring = 1.

***p < .001.

The final model examining predictors of code-switching during the Spanish task revealed several significant effects (see Table 5 for full results). Children in first grade (*b* = −0.20, *SE* = 0.09, *z* = −2.2, *p* = .028) and second grade (*b* = −0.28, *SE* = 0.11, *z* = −2.66, *p* = .008) showed significantly lower rates of code-switching from Spanish to English on the Spanish task compared with kindergartners. Code-switching also decreased in the Spring compared with the Fall semester (*b* = −0.22, *SE* = 0.08, *z* = −2.96, *p* = .003). Notably, longer English MLU was associated with increased switching from Spanish to English on the Spanish task (*b* = 0.16, *SE* = 0.04, *z* = 3.75, *p* < .001), suggesting that greater English proficiency may drive children to revert to English when speaking in Spanish. In contrast, a higher NDW in Spanish predicted lower rates of code-switching (*b* = −0.006, *SE* = 0.00, *z* = −2.31, *p* = .021), indicating that stronger Spanish lexical diversity may keep children from code-switching to English when the task is in Spanish. Gender was also a small but significant predictor, with boys showing slightly higher rates of code-switching than girls (*b* = 0.32, *SE* = 0.16, *z* = 2.00, *p* = .046). No significant effects were found for Spanish MLU or English NDW.

**Table 5:** Poisson generalized linear mixed-effects model predicting code-switching count on the Spanish task based on language proficiency.

Fixed effects				
Predictor	Estimate (*b*)	*SE*	*z*	*p*
(Intercept)	−5.28	0.3	−17.46	<.001***
Gender (Male)	0.32	0.16	2	.046*
Grade 1	−0.2	0.09	−2.2	.028*
Grade 2	−0.28	0.11	−2.66	.008**
Spring (vs. Fall)	−0.22	0.08	−2.96	.003**
MLU (English)	0.16	0.04	3.75	<.001***
MLU (Spanish)	0.06	0.05	1.2	.232
NDW (English)	0	0	1.24	.214
NDW (Spanish)	−0.01	0	−2.31	.021*

Random effects		
Group	Variance	*SD*
Subject (Intercept)	0.90	0.95

Model fit statistics	
Statistic	Value
AIC	3,158.6
BIC	3,209.2
Log Likelihood	−1,569.3
Deviance	3,138.6

*Note*. The model was fit using the Poisson distribution with a log link and included an offset for total utterances (log_Utts). Gender was coded as Female = 0, Male = 1 and wave as Fall = 0, Spring = 1.

^*^ *p* < .05, **p < .01, ***p < .001.

Given that a large proportion of children did not engage in any code-switching (see Table 2), we ran the same analyses as reported above with a sub-sample of children who code-switched on each task (i.e., removing any instance where no code-switching occurred; *N* = 141). When evaluating performance, when children were asked to narrate the story in English, the previous main pattern of code-switching across grade and wave remained, as did the relation between higher NDW in English and less code-switching from English to Spanish (see Table 6 for full statistics). Interestingly, the relation between NDW in Spanish and code-switching from English to Spanish on the English task was no longer significant. Instead, MLU emerged as a significant predictor of code-switching from English to Spanish: a smaller MLU in English and a larger MLU in Spanish were associated with a higher likelihood of code-switching from English to Spanish.

**Table 6:** Poisson generalized linear mixed-effects model predicting code-switching count on the English task based on language proficiency among children who code-switched.

Fixed effects				
Predictor	Estimate (*b*)	*SE*	*z*	*p*
(Intercept)	−0.95	0.34	−2.79	.01*
Gender (Male)	−0.02	0.17	−0.11	.92
Grade 1	−1.00	0.11	−9.44	<.001***
Grade 2	−1.33	0.16	−8.31	<.001***
Spring (vs. Fall)	−0.22	0.10	−2.28	.02*
MLU (English)	−0.15	0.05	−3.00	.002**
MLU (Spanish)	0.20	0.07	2.96	.003**
NDW (English)	−0.01	0.00	−4.50	<.001***
NDW (Spanish)	0.00	0.00	−0.09	.93

Random effects		
Group	Variance	*SD*
Subject (Intercept)	0.61	0.78

Model fit statistics	
Statistic	Value
AIC	1,401.2
BIC	1,437.4
Log Likelihood	−690.6
Deviance	1,381.2

*Note*. The model was fit using the Poisson distribution with a log link and included an offset for total utterances (log_Utts). Gender was coded as Female = 0, Male = 1 and wave as Fall = 0, Spring = 1.

*p < .05, **p < .01, ***p < .001.

When evaluating performance, when children were asked to narrate the story in Spanish, the previous main effect of Spanish NDW remained significant; however, no other associations remained significant (see Table 7 for full statistics).

**Table 7:** Poisson generalized linear mixed-effects model predicting code-switching count on the Spanish task based on language proficiency among children who code-switched.

Fixed effects				
Predictor	Estimate (*b*)	*SE*	*z*	*p*
(Intercept)	−3.10	0.27	−11.63	<.001***
Gender (Male)	0.09	0.10	0.90	.37
Grade 1	0.12	0.10	1.20	.23
Grade 2	−0.16	0.11	−1.50	.13
Spring (vs. Fall)	−0.06	0.08	−0.81	.42
MLU (English)	0.06	0.04	1.49	.14
MLU (Spanish)	0.07	0.05	1.43	.15
NDW (English)	0.00	0.00	0.92	.36
NDW (Spanish)	−0.01	0.00	−4.54	<.001***

Random effects		
Group	Variance	*SD*
Subject (Intercept)	0.21	0.46

Model fit statistics	
Statistic	Value
AIC	1,813.6
BIC	1,855.0
Log Likelihood	−896.8
Deviance	1,793.6

*Note*. The model was fit using the Poisson distribution with a log link and included an offset for total utterances (log_Utts). Gender was coded as Female = 0, Male = 1 and wave as Fall = 0, Spring = 1.

***p < .001.

## Discussion

Despite the various methodological differences in the age of participants, language elicitation methods, and environmental contexts, the code-switching literature is consistent in the fact that not all bilingual children code-switch and that code-switching is more frequent when children are speaking their nondominant language. Code-switching can be observed in very young children (Montanari et al., 2019; Smolak et al., 2019) and continues to be evident in school-age children (Greene et al., 2013), provided that both languages are used in the child's environment. Engaging in a task that does not constrain the use of code-switching, such as interacting in a conversational task with a bilingual interlocutor, facilitates the use of code-switching (Gross & Kaushanskaya, 2022). In contrast, monoglossic tasks such as producing oral story narratives reduce the amount of code-switching (Gutierrez-Clellen et al., 2009). Thus, the specific communicative task, conversation versus story narration, might either foster or constrain code-switching. The type of program the child is enrolled in should also contribute to the amount of code-switching. For example, regardless of task, one would expect to observe more code-switches among children enrolled in a two-way bilingual program than among children in an English immersion program, since the use of both languages is more acceptable in the former.

Our present study adds to the existing literature in a couple of ways. First, the participants in this study, although linguistically heterogeneous with respect to the relative strength of their Spanish and English (Su et al., 2022), all entered Kindergarten with limited English skills. This allowed us to examine the possible shifts in code-switching as a function of changes in relative proficiency in their languages. Furthermore, all of the children were enrolled in an educational program in which all instruction was in English, with little or no Spanish used during the instructional period. Based on the learning environment, we expected that as the children's proficiency in English increased, the amount of code-switches on the English task would decrease, and that the amount of code-switches on the Spanish task would be stable or slightly increase.

We first examined whether bilingual children demonstrate asymmetries in their code-switching and how these asymmetries change as children progress through school. Our original intent was to examine intrasentential and intersentential code-switching. However, the use of intersentential code-switching was minimal and only occurred in a few situations in which children engaged in conversations with the examiners, usually asking for clarifications. Thus, the focus of this study centered on intrasentential code-switching. Consistent with the literature (Gross & Castilla-Earls, 2023; Gutierrez-Clellen et al., 2009), not all children code-switched. Some children did not code-switch in either language, some just in one language, and some in both. While the number of children at school entry who code-switched across language tasks was similar (66 on the English task compared with 68 on the Spanish task), by the end of second grade, significantly more children code-switched on the Spanish task (11 on the English task compared with 112 on the Spanish task). This finding provides some insight into the reported variability in the proportion of children who code-switch, and in what language they code-switch, emphasizing the need to examine both languages using multiple-year longitudinal data. Doing otherwise would only provide a snapshot of random points in the developmental process.

At kindergarten entry, bilingual children were far more likely to code-switch from English to Spanish when completing the task in English than to code-switch from Spanish to English when narrating the story in Spanish. Although this pattern holds steady in the Fall of each grade, as children progress through school, we observed that code-switching behaviors shifted, and bilingual children were more likely to code-switch from Spanish to English when completing the Spanish task. Importantly, these patterns of code-switching behavior were related to children's language proficiency, as measured by MLU and NDW: when children demonstrated greater language proficiency in Spanish and weaker language proficiency in English, they were more likely to code-switch from English to Spanish on the English task, but as children developed greater proficiency in English and weaker proficiency in Spanish, they were more likely to code-switch from Spanish to English on the Spanish task. These patterns suggest that the use of code-switching parallels shifts in relative proficiencies, which were expected due to their enrollment in a program that focused solely on English.

When focusing only on children who code-switched, several patterns observed in the full sample shifted. On the English task, the previously significant relation between Spanish NDW and code-switching from English to Spanish was no longer evident. Instead, MLU emerged as a robust predictor: children with shorter MLUs in English and longer MLUs in Spanish were more likely to switch into Spanish, suggesting that expressive grammatical skills in each language shape children's switching behavior. On the Spanish task, the positive association between English MLU and switching from Spanish to English remained significant in the full sample but disappeared in the subset of children who code-switched. The only consistent predictor across both samples was Spanish NDW, which continued to predict switching from Spanish to English—indicating that broader Spanish vocabulary may help children maintain the target language. One possible explanation for these differences is reduced variability in the code-switching-only group, which may attenuate weaker effects. It may also be that different mechanisms support whether a child code-switches versus how much they code-switch once they do—­highlighting the value of separating predictors of initiation from those of frequency.

Our findings suggest that children's language environments across the academic year influence not only their overall language proficiency but also their code-switching behavior. Specifically, children were more likely to code-switch from English to Spanish in the Fall, while rates of switching from Spanish to English remained more stable across semesters. This asymmetry points to shifts in relative language proficiency driven by environmental exposure: during summer break, children likely spend more time in Spanish-speaking home environments, which strengthens their Spanish skills while English input diminishes. This phenomenon, often referred to as the “summer slump”, is well-documented. For example, Rojas and Iglesias (2013) found that children's English skills consistently decreased during the summer, while their Spanish skills continued to increase. Similarly, Scheffner Hammer et al. (2008) demonstrated that preschool-aged bilingual children's English receptive language scores improve throughout their first year in Head Start but decline significantly during summer break, before increasing again in their second year of Head Start. Importantly, these semester effects on code-switching varied by grade and task language: in first grade, the Spring decline in code-switching was attenuated during the Spanish task, whereas in second grade, this pattern reversed—children showed significantly more code-switches from Spanish to English in the Spring. This developmental shift suggests that by second grade, increased exposure to English in school may lead children to default to English even during Spanish tasks. Therefore, children's primary language environments likely impact their relative proficiency in their two languages and influence their code-switching behaviors during narrative retell, with (i) the home language environment influencing their code-switching behavior in the Fall, and (ii) the school environment influencing their code-switching by the Spring. Throughout the period assessed, whether due to a lexical gap (Tulloch & Hoff, 2023) or lexical competition (Sarkis & Montag, 2021), children code-switched to maximize their communicative power.

There are some limitations that should be considered when interpreting these results. First, the context in which code-switching was measured should be considered. In the current study, we utilized a narrative retell paradigm that was not explicitly designed to measure code-switching. We predict that, in social interactions that are less structured, children likely code-switch more. However, the use of a more structured paradigm speaks to the robustness of our findings, as we would anticipate dampened code-switching effects using this paradigm compared with more naturalistic measures. Second, since children were specifically instructed to complete the task in English or in Spanish, this may have restricted their likelihood to code-switch. In other words, it is unclear whether asking children to complete the task in any language would have led to a greater amount of code-switching. Future work should examine this possibility. Third, the order of language elicitation was fixed in the current study, with all children narrating the story first in Spanish and then in English 1 week later. This consistent sequencing may have influenced how children encoded or recalled the story, particularly given their growing bilingual proficiency over time. While this approach was chosen to ensure was to ensure that kindergarten children who were coming from predominantly Spanish-speaking homes were more comfortable completing the task in their home language and to maintain consistency across waves, it may have introduced an order effect that could have impacted narrative performance. Future studies should consider counterbalancing language order to disentangle potential sequencing effects. Fourth, children were tested in schools where English was the dominant language, but came from homes where English was not frequently used. Although we would anticipate increased code-switching from Spanish to English due to these location differences, a finding which was not explored in this study, we cannot definitively rule out the possibility that our data reflect more temporary, context-dependent changes (Oppenheim et al., 2020). Fifth, research has shown that children who hear or use a language more frequently do not always have higher proficiency in that language (Bedore et al., 2012). For this reason, we measured language proficiency as both the difference in the NDW spoken and the mean length of utterance in Spanish versus English during the task. However, measuring children's language proficiency more globally, by using standardized measures of receptive and expressive skills—or measures of real-time language use outside of a given task, may provide a more comprehensive understanding of the relation between language proficiency and code-switching.

Extensive bilingual research has indicated that an individual's proficiency in their second learned language (L2), if spoken by the societal majority, often surpasses their proficiency in their first learned language (L1; see Oller et al., 2011, for a review). The current study bolsters these findings, as children's language proficiency in both their L1 and L2 influenced code-switching behavior from grades K-2. Specifically, when children had higher levels of Spanish proficiency, they were more likely to code-switch from English to Spanish, but as children became more proficient in their L2, English, their likelihood of code-switching from English to Spanish declined, while their likelihood of code-switching from Spanish to English increased.

Multilingual populations in the US have seen substantial growth over the past several decades (Dietrich & Hernandez, 2022). Although not yet the majority of the population within the US, bi- and multilingualism represent the majority of children globally. As of 2019, more than 11.2 million young children, or 33% of all U.S. children under the age of 9, are bilingual (Migration Policy Institute, 2019). Many bilingual children show lower levels of school achievement in the U.S. education system: 31% fail to complete high school—compared with 10% for monolinguals (August et al., 2009). While the implications of these statistics should not be overlooked, our data suggest that supporting bilingual children's proficiency in both languages over the course of the summer might help to shape their patterns of language use in school. Further, it is possible that assessing bilingual children's language skills at the start of the Fall semester may be misrepresentative and rooted in the linguistic context in which the child has spent the summer months. To advance theories of bilingual language development and guide educational, clinical, and policy decisions, it is essential to consider not only how language skills are assessed, but also when—ensuring that assessment tools and timing accurately reflect the dynamic linguistic experiences of bilingual children (e.g., Greene et al., 2013). The findings of the current study contribute to this mission by characterizing common patterns of code-switching practices used by bilingual children as they progress through school.

## Data Availability

Output data from SALT, demographic data, and the analytic code necessary to reproduce the analyses presented in this paper are publicly accessible at the following URL: https://github.com/juliagoolia28/manuscripts/tree/master/code_switch_morini. Raw data (language transcripts) and the materials necessary to replicate the findings presented here are not publicly accessible. The analyses presented here were not preregistered. Predicted rate of code-switching per utterance across task language (English, Spanish), wave (Fall, Spring), and grade (kindergarten, first, second). *Note*. Predicted values were derived from a Poisson generalized linear mixed-effects model with an offset for total utterances. The model included fixed effects for Language, Wave, and Grade, and their interactions, and Gender, as well as a random intercept for subject. The *y*-axis reflects the expected number of code-switches per utterance. Error bars represent 95% confidence intervals.

## Footnotes

1 Output data from SALT, demographic data, and the analytic code necessary to reproduce the analyses presented in this paper are publicly accessible at the following URL: https://osf.io/k4gny/?view_only=57831061d54f460e9ff2a817efeebbf2. Raw data (language transcripts) and the materials necessary to replicate the findings presented here are not publicly accessible. The analyses presented here were not preregistered.
